# Interaction of Polyamines, Abscisic Acid, Nitric Oxide, and Hydrogen Peroxide under Chilling Stress in Tomato (*Lycopersicon esculentum* Mill.) Seedlings

**DOI:** 10.3389/fpls.2017.00203

**Published:** 2017-02-14

**Authors:** Qiannan Diao, Yongjun Song, Dongmei Shi, Hongyan Qi

**Affiliations:** ^1^College of Horticulture, Shenyang Agricultural UniversityShenyang, China; ^2^Key Laboratory of Protected Horticulture of Ministry of Education and Liaoning Province, Collaborative Innovation Center of Protected Vegetable Surround Bohai Gulf RegionShenyang, China

**Keywords:** polyamines, hydrogen peroxide, nitric oxide, abscisic acid, tomato, chilling stress

## Abstract

Polyamines (PAs) play a vital role in the responses of higher plants to abiotic stresses. However, only a limited number of studies have examined the interplay between PAs and signal molecules. The aim of this study was to elucidate the cross-talk among PAs, abscisic acid (ABA), nitric oxide (NO), and hydrogen peroxide (H_2_O_2_) under chilling stress conditions using tomato seedlings [(*Lycopersicon esculentum* Mill.) cv. Moneymaker]. The study showed that during chilling stress (4°C; 0, 12, and 24 h), the application of spermidine (Spd) and spermine (Spm) elevated NO and H_2_O_2_ levels, enhanced nitrite reductase (NR), nitric oxide synthase (NOS)-like, and polyamine oxidase activities, and upregulated *LeNR* relative expression, but did not influence *LeNOS1* expression. In contrast, putrescine (Put) treatment had no obvious impact. During the recovery period (25/15°C, 10 h), the above-mentioned parameters induced by the application of PAs were restored to their control levels. Seedlings pretreated with sodium nitroprusside (SNP, an NO donor) showed elevated Put and Spd levels throughout the treatment period, consistent with increased expression in leaves of genes encoding arginine decarboxylase (*LeADC. LeADC1*), ornithine decarboxylase (*LeODC*), and Spd synthase (*LeSPDS*) expressions in tomato leaves throughout the treatment period. Under chilling stress, the Put content increased first, followed by a rise in the Spd content. Exogenously applied SNP did not increase the expression of genes encoding *S*-adenosylmethionine decarboxylase (*LeSAMDC*) and Spm synthase (*LeSPMS*), consistent with the observation that Spm levels remained constant under chilling stress and during the recovery period. In contrast, exogenous Put significantly increased the ABA content and the 9-*cis*-epoxycarotenoid dioxygenase (*LeNCED1*) transcript level. Treatment with ABA could alleviate the electrolyte leakage (EL) induced by D-Arg (an inhibitor of Put). Taken together, it is concluded that, under chilling stress, Spd and Spm enhanced the production of NO in tomato seedlings through an H_2_O_2_-dependent mechanism, via the NR and NOS-like pathways. ABA is involved in Put-induced tolerance to chilling stress, and NO could increase the content of Put and Spd under chilling stress.

## Introduction

Chilling stress is a critical factor that can limit crop productivity, since it can affect a range of physiological processes in plants and can cause both metabolic disruption and structural damages (Atıci and Nalbantoǧlu, 2003). Therefore, plants have developed a number of strategies to cope with chilling stress, some of which involve the accumulation of ABA (Anderson et al., 1994) and polyamines (PAs) (Shen et al., 2000; Groppa and Benavides, 2008).

Polyamines, principally Put, Spd, and Spm, are low-molecular-weight aliphatic amine compounds that are ubiquitous in all plant cells and that participate in a range of cellular processes, including cell division and elongation, morphogenesis, flowering, senescence, and seed germination (Bais and Ravishankar, 2002; Kusano et al., 2007; Gupta et al., 2013). In plants, Put can be synthesized via the decarboxylation of arginine by ADC (EC 4.1.1.19) or of ornithine by ODC (EC 4.1.1.17). Spd and Spm are formed by the addition of an aminopropyl moiety in reactions catalyzed, respectively, by SPDS (EC 2.5.1.16) and SPMS (EC 2.5.1.12). The aminopropyl groups are contributed by decarboxylated *S*-adenosylmethionine (dc-SAM), which is derived from *S*-adenosylmethionine (SAM) by the action of SAMDC (EC 4.1.1.50). As has been well documented, DAO (EC 1.4.3.6) and PAO (EC 1.5.3.3) are responsible for the oxidation of PAs in plant cells, which occurs with the concomitant production of H_2_O_2_ (Martin-Tanguy, 2001). It has been suggested PAs are essential for improving stress tolerance in various plants (Durmus and Kadioglu, 2005; Duan et al., 2008; Arasimowicz-Jelonek et al., 2009; Hu et al., 2012; Li et al., 2014; Minocha et al., 2014). PAs can function directly as protective compounds, thereby enhancing plant tolerance to abiotic stresses; because of their polycationic nature at physiological pH, PAs can interact with negatively charged macromolecules in the cell membrane and can thereby stabilize cell membrane structure under stress conditions (Groppa and Benavides, 2008; Alcázar et al., 2010). In addition to this directly protective role, PAs also interact with other protective molecules, such as ABA, SA, GABA, and BR, through which they can also modulate stress tolerance (Iqbal et al., 2006; Alcázar et al., 2010; Wang and Zhang, 2012; Espasandin et al., 2014; Hatmi et al., 2015; Serna et al., 2015). Furthermore, PAs may be involved in secondary messenger signaling cross-talk (Alcázar et al., 2010). Recent reports have indicated that PAs are associated with the production of H_2_O_2_ and NO (Tun et al., 2006; Angelini et al., 2008; Groppa et al., 2008; Hussain et al., 2011; Yang et al., 2014).

Nitric oxide is a highly reactive gaseous molecule that regulates a diverse range of physiological processes, including germination, metabolism, transport, flowering, and senescence (Neill et al., 2003; Besson-Bard et al., 2008). In addition, the available evidence indicates that NO is an important signal molecule involved in multiple plant responses toward a variety of abiotic and biotic stresses (García-Mata and Lamattina, 2001, Hung et al., 2002; Besson-Bard et al., 2008). It is well-established that the exogenous application of NO can protect plants against stress conditions by promoting growth, PS II) activity, and the maintenance of ionic homeostasis, as well as by activating antioxidant enzymes (Uchida et al., 2002; Shi et al., 2005; Zhang et al., 2006; Ziogas et al., 2015). Furthermore, NO acts in concert with other plant growth regulators, such as ABA and PAs (García-Mata and Lamattina, 2001, 2002; Fan et al., 2013; Filippou et al., 2013; Gong et al., 2014); and in *Arabidopsis*, cucumber, wheat, and soybean, it has been proposed that PAs are able to induce NO generation (Tun et al., 2006; Groppa et al., 2008; Yang et al., 2014). Thus, under conditions of environmental stress, there may be a link between PAs and NO; and this may involve ABA as well.

Several potential NO sources exist in plants, notably the widely known NR and NOS pathway (Desikan et al., 2002; Guo et al., 2003). NR catalyzes the reduction of nitrate to nitrite, using NADH as principal electron donor (Gupta et al., 2011), and this activity is widespread or ubiquitous in higher plants (Desikan et al., 2002; Rockel et al., 2002; Dordas et al., 2003). NOS is responsible for NO synthesis in animals. Although mammalian-type NOS is intricate and a corresponding activity in plants has yet to be fully characterized (Guo et al., 2003; Zemojtel et al., 2006), NOS-like activity has been detected extensively in plants. Moreover, inhibitors of mammalian NOS can inhibit NO production in plants (Neill et al., 2008; Tewari et al., 2013).

Another important signal molecule, H_2_O_2_, is also capable of participating in several physiological processes in plants, including adaptive stress responses (Finkel and Holbrook, 2000; Neill et al., 2003; Bright et al., 2006; Dickinson and Chang, 2011). NADPH oxidases, apoplast amine oxidases, and oxalate oxidase are the main enzymatic sources for the generation of reactive oxygen species (ROS) (Grant and Loake, 2000; Mittler, 2002; Cona et al., 2006). PAs may either trigger ROS synthesis or scavenge ROS, depending on the concentrations of intracellular PAs (Saha et al., 2015). In many cases, the production of H_2_O_2_ via the PA catabolic pathway serves as a protective measure; this process has been well documented (Walters, 2003; Cona et al., 2006). Recent reports also show a relationship between NO and H_2_O_2_. Depending upon the stress conditions, the generation of NO and H_2_O_2_ may occur either in parallel, or in rapid succession (de Pinto et al., 2002; Bright et al., 2006; Pasqualini et al., 2009).

Abscisic acid is one of the most important phytohormones involved in plant growth, development, and adaptation to a range of stress conditions (Schroeder et al., 2001; Verslues et al., 2006). It has been proposed that 9-*cis*-epoxycarotenoid dioxygenase (NCED) is the rate-limiting enzyme in ABA synthesis (Qin and Zeevaart, 1999; Jahromi et al., 2008). It is well established that PAs, ABA, NO, and H_2_O_2_ are, from a functional perspective, multifaceted molecules, involved in several stress responses in many plant species (Shen et al., 2000; He et al., 2002; Zheng et al., 2009; Yang et al., 2011; Yamamoto et al., 2012; Wang et al., 2013). In our previous study, we observed that both Put and Spd enhanced the chilling tolerance of tomato seedlings (Song et al., 2014b; Diao et al., 2015). It is notable that PAs, ABA, NO, and H_2_O_2_ participate in various physiological and stress responses via a complex network, and that there is an intricate association between them during a plant’s response to abiotic stress (Wimalasekera et al., 2011b). To our knowledge, only a small number of studies have examined the potential links between PAs and signal molecules, and PAs and ABA in response to chilling stress in tomato seedlings. Therefore, in this study, we analyzed the effect of PAs on NO and H_2_O_2_ synthesis in tomato seedlings under chilling stress. At the same time, we examined the changes in PA levels, PA biosynthetic enzymes, and relative gene expression triggered by NO. Furthermore, we investigated the influence of PAs on ABA production under chilling stress.

## Materials and Methods

### Plant Materials, Growth, and Treatment Conditions

Plants were grown at Shenyang Agricultural University, Shenyang, China. Seeds of tomato (*Lycopersicon esculentum* Mill. cv. Moneymaker) were germinated in 50-hole plates containing peat moss. When the seedlings had developed two true leaves, they were transplanted into 12 cm × 12 cm plastic trays filled with peat moss in a greenhouse (average day/night temperature, 25°C/15°C) with natural light at a relative humidity of 60% and watered daily. Seedlings were used for experiments at the five-leaf stage.

To investigate the effects of exogenous PAs, seedlings were subjected to four treatments: (1) H_2_O+chilling (as control); (2) 1 mM Put+chilling; (3) 1 mM Spd+chilling; and (4) 1 mM Spm+chilling. The leaves of the tomato seedlings were sprayed completely with 1 mM Put, 1 mM Spd, or 1 mM Spm once each day for a week. Following transfer to a phytotron, the seedlings were then subjected to chilling stress. The environmental conditions were: photosynthetic photon flux density (PPFD), 600 μmolm^-2^s^-1^; temperatures, 4°C. Samples were taken at 0, 12, and 24 h for physiological and biochemical analyses (including determinations of NO and H_2_O_2_ levels, DAO and PAO activities, NR activity, NOS-like activities, and relative gene expressions). After 24 h of chilling stress treatment, seedlings were maintained under 25°C/15°C conditions for a further 10 h for recovery.

In order to investigate the relationship between NO and H_2_O_2_ induced by Spd, before chilling treatment (4°C), some seedlings were treated by spraying with 200 μM N^G^-nitro-L-arginine methyl ester HCl (L-NAME, an inhibitor of NOS), 200 μM tungstate (an inhibitor of NR), and 200 μM PTIO (2-phenyl-4,4,5,5-tetramethylimidazoline-1-oxyl-3-oxide, a scavenger of NO) prior to the Spd+chilling treatment. Other seedlings were treated by spraying with 100 μM DPI (2,6-DPI, a NADPH oxidase inhibitor) and 5 mM DMTU (dimethylthiourea, a H_2_O_2_ and OH⋅ scavenger), In each case, the pre-treatment was carried out for 12 h daily (from 18:00 until 06:00) for 3 days. The seedlings were then sprayed with 1 mM Spd, 12 h after the final treatment. Seedlings sprayed with distilled water and subjected to chilling at 4°C and at a PPFD of 600 μmolm^-2^s^-1^ served as the control. Leaves were harvested for NO and H_2_O_2_ analyses 24 h after the chilling treatments.

To investigate whether NO induced PAs, prior to chilling stress treatment seedlings were sprayed with 200 μM SNP (a NO donor). Leaves were then harvested following 0, 12, and 24 h of chilling treatment, and in addition after 10 h of recovery at 25°C/15°C, for determination of PAs and analyses of relative gene expression.

To investigate the effect of Put on endogenous ABA, prior to chilling stress treatment (4°C), seedlings were sprayed with 1 mM D-arginine (D-Arg) (an inhibitor of Put synthesis), and then treated with Put or distilled water 12 h later. Leaves were harvested following 0, 12, and 24 h of chilling treatment, for analyses of ABA levels and *LeNCED1* expression.

To investigate the effect of ABA on EL, seedlings were sprayed with 1 mM ABA and 1 mM D-Arg, separately, and then 12 h afterward were exposed to chilling stress (4°C) for 24 h. Other seedlings were sprayed with 1 mM D-Arg alone; after 12 h, these were then sprayed with 1 mM ABA and exposed to chilling stress for 24 h.

For each of these treatments, seedlings sprayed with distilled water in place of the respective reagents, but otherwise treated identically, served as controls.

For all treatments described, the third and fourth fully expanded leaves from 12 uniform seedlings were used for analysis. The leaves were repeatedly washed in deionized distilled water, and then frozen in liquid nitrogen and stored at -80°C prior to analysis.

### PA Determination

Free PAs were quantified by the method of Duan et al. (2008), with slight modifications. Leaves (0.5 g) were homogenized in 3 mL of chilled 0.5% (w/v) perchloric acid (PCA), kept on ice for 1 h, and then centrifuged at 12,000 × *g* and 4°C for 20 min. The supernatant (500 μL) was then mixed with 1 mL of 2 M NaOH and 7 μL of benzoyl chloride. The mixture was vortexed for 20 s, and then incubated for 30 min at 37°C. Saturated NaCl (2 mL) was then added and the benzoyl PAs were extracted with 2 mL of diethyl ether. The sample was then centrifuged at 1,500 × *g* for 5 min. Finally, 1 mL of the ether phase was taken, the ether was evaporated and the residue was re-dissolved in 100 μL of methanol. PA standards were prepared as described for leaf samples. The content of PAs was analyzed by HPLC (Waters 600, Waters Co., Ltd, USA). PAs were separated on a reverse-phase C-18 column eluted at room temperature using 60% (v/v) methanol at a flow rate of 0.8 mL/min. The absorbance at 254 nm was measured using a UV–vis detector.

### ABA Determination

Abscisic acid was extracted and measured according to Lopez-Carbonell and Jauregui (2005). Leaf samples (0.5 g) were homogenized at -20°C in acetone:water:acetic acid (80:19:1, v/v), vortexed, and centrifuged at 10,000 × *g* and 4°C for 10 min. The supernatants were collected and the pellets re-extracted with extraction solvent. The second extraction was centrifuged, and then the first and second supernatants were combined and dried under a nitrogen stream. The dried samples were kept at -20°C. Immediately prior to analysis, the extracts were re-dissolved in 200 μL of water:acetonitrile:acetic acid (90:10:0.05, v/v/v), stirred, vortexed, cleared by centrifugation at 10,000 × *g* for 5 min, and then filtered through a 0.45-μm polytetrafluoroethylene filter. ABA was determined by HPLC (Waters 600, Waters Co., Ltd, USA) at 30°C, using a 4.6-mm × 250-mm reverse-phase C-18 column, with elution by a linear gradient of 0.05% (w/v) acetic acid in water (solvent A) and acetonitrile at a flow rate of 0.4 ml/min^-1^. The gradient profile [*t*(min), % A] was: (0, 85), (5, 0), (5.2, 0), (6, 85), (10, 85). The absorbance at 262 nm was measured using a UV–vis detector.

### Determination of NO

Nitric oxide content was determined as described by Murphy and Noack (1994), with some modifications. Leaf samples (0.5 g) were incubated for 5 min with 100 U of catalase and 100 U of superoxide dismutase to remove endogenous ROS, prior to the addition of 10 mL of 5 mM oxyhemoglobin (HbO_2_). After 2 min, NO was determined spectrophotometrically by measuring the conversion of HbO_2_ to methemoglobin (metHb), and the NO content was calculated using an extinction coefficient of 77 mM^-1^ cm^-1^ [*A*_401_ (metHb)-*A*_421_ (HbO_2_)].

### Fluorescent Detection of NO

Nitric oxide was detected using the fluorescent dye DAF-FM DA (Sigma). Epidermal fragments of tomato were placed in 1 ml of DAF-FM DA buffer solution (10 mM Tris–HCl, pH 7.2) and were then incubated for 20 min at room temperature with 1 ml of 5 μM DAF-FM DA in 10 mM Tris–HCl buffer (pH 7.2). The incubation solutions were then pipetted off. After washing with fresh loading buffer three times, the epidermal fragments were mounted on a microscope slide in the same medium for examination with a Zeiss Axiovert 200 M inverted microscope equipped with a confocal laser scanner (Zeiss LSM 510). Excitation and emission were at 495 and 515 nm, respectively. Images were processed and analyzed using Zeiss 2012 software.

### Determination of H_2_O_2_

Hydrogen peroxide content was quantified by the method of Patterson et al. (1984), with some modifications. Leaf samples (0.5 g) were homogenized in 3 mL of ice-cold acetone. Titanium reagent (20% v/v titanic tetrachloride in concentrated HCl) was then added to 1 mL of extract supernatant. The Ti-H_2_O_2_ complex was then precipitated by adding 0.2 mL of 17 M ammonia solution and centrifuging at 30,000 × *g*, for 10 min at 4°C, the supernatant being discarded. The pellet was washed three times by centrifugation and resuspension in ice-cold acetone, and finally dissolved in 3 mL of 1 M H_2_SO_4_. The absorbance of the solution was measured at 410 nm and an extinction coefficient of 0.28 mM^-1^ cm^-1^ was used for calculation.

### Histochemical Detection of H_2_O_2_

Hydrogen peroxide was detected visually by using 3,-3-diaminobenzidine (DAB) staining method according to Orozoco-Cardenas and Ryan (1999), with some modifications. Leaves of seedlings subjected to different treatments were immersed in a 1 mg mL^-1^ solution of DAB (Sigma) and then vacuum-infiltrated for 30 min. After incubation for 9 h at 25°C, the leaves were decolorized in 95% ethanol at 80°C for 30 min, and then photographed.

### Determination of DAO and PAO Activities

Diamine oxidase and PAO activities were measured by the method of Song et al. (2014a). Leaf samples (0.5 g) were homogenized in chilled potassium phosphate buffer (0.1 mol⋅L^-1^, pH 6.5). The homogenate was centrifuged at 10,000 × *g*, for 20 min at 4°C. The supernatant was then used for the assay of DAO and PAO.

### Determination of NR Activity

Nitrite reductase activity was determined according the method of Scheible et al. (1997), with some modifications. Leaf samples (0.5 g) were first homogenized in chilled extraction buffer comprising 100 mM HEPES-KOH, pH 7.5, 5 mM dithiothreitol, 1 mM EDTA, 10% (v/v) glycerol, 0.1% (w/v) Triton X-100, 0.5 mM PMSF, 1 μM leupeptin, 20 μM flavin adenine dinucleotide (FAD), 5 μM Na_2_MoO_4_, and 1% (w/v) PVPP, and then centrifuged at 10,000 × *g* and 4°C for 20 min. One volume of supernatant was then mixed with five volumes of assay buffer (100 mM HEPES-KOH, pH 7.5, 5 mM KNO_3_, and 0.25 mM NADH). The reaction was started by the addition of assay buffer, incubated at 25°C for 30 min, and then stopped by adding 0.1 M zinc acetate. After 15 min, the stopped reaction mixture was centrifuged at 13,000 × *g* for 5 min. The supernatant was then added to sulfanilamide (1%, v/v, in 3 M HCl) and *N*-1-naphthylethylenediamine (0.02%, w/w). The nitrite produced was measured colorimetrically at 520 nm.

### Determination of NOS-Like Activity

The NOS-like activity was assayed using a NOS colorimetric assay kit (Nanjing Jiancheng Bioengineering Institute, Jiangsu, China). Leaf samples (0.5 g) were homogenized in 2 mL of 50 mM phosphate-buffered saline, pH 7.4, containing 1.0 mM leupeptin, 1.0 mM EDTA, 10.0 mM ethyleneglycol bis (2-aminoethyl ether) tetraacetic acid, 1.0 mM PMSF and 10 g L^-1^ PVPP. After centrifuging at 15,000 × *g* for 20 min, the supernatant was incubated with the kit assay reagent for 15 min at 37°C and the reaction was then terminated using the stop buffer from the NOS assay kit. The absorbance was measured at 530 nm.

### Determination of Electrolyte Leakage

Electrolyte leakage was determined according to Sairam and Srivastava (2002). Leaf samples (0.2 g) were rinsed three times with deionized water, and then kept in 20 ml of distilled water at 25°C for 3 h. The electrical conductivity of the solution (initial electrical conductivity E1) was then measured. The solution containing the leaves was then boiled at 100°C for 30 min to release all electrolytes, cooled to 25°C, and the final electrical conductivity (E2) was measured. The relative EL was calculated as E1/E2 and expressed as a percentage.

### Total RNA Extraction and Gene Expression Analysis

Total RNA was extracted using an RNAprep pure plant total RNA extraction kit (Kangwei, Beijing, China). DNA contamination was eliminated by adding RQ1 DNAse (Promega). Total RNA concentrations of samples were routinely determined from *A*_260_ and *A*_280_ values and integrity was checked by agarose gel electrophoresis.

RNA samples were reverse-transcribed into cDNAs according to the kit manufacturer’s instructions (Tiangen Biotech Co. Ltd, Beijing, China). The resulting cDNAs were used as templates for PCR amplification. To confirm accuracy, all PCR products of the expected size were sequenced (Sangon Biotech Co. Ltd, Shanghai, China).

Primers were designed using Primer Express 3.0 software. The PCR primer sequences are listed in **Table 1**. Real-time PCR analysis was performed as follows. The cDNA samples were used as the template and mixed with 200 nmol of each primer and SYBR Green PCR Real Master Mix (Tiangen Biotech Co. Ltd, Beijing, China) for real-time PCR analysis using an ABI 7500 (Applied Biosystems, USA). To determine relative gene expression for each sample, the threshold cycle (*C*_t_) value was normalized to *actin* and set relative to control samples according to the 2^-ΔΔ^*^C^*^t^ method.

**Table 1:** Nitric oxide-interrelated genes and PAs biosynthetic enzyme genes accession numbers and primer sequences of the genes described in this study.

Category	Accession	Encode corresponding enzyme	Primer sequences (5′–3′)
*LeADC*	HM629957	ADC	F5′- TGCTTGAAGTGTCTCTTG -3′R5′- GATTGCGGTCATAACATAAG -3′
*LeADC1*	NM_001247135	ADC	F5′- CACAAGGAAGAAGAAGTAGA -3′R5′- GCCAACACCAACAATATTC -3′
*LeODC*	NM_001247687	ODC	F5′- TAAGGGATTACCAGTTACC -3′R5′- GGATAAGCATAAGCAAGG -3′
*LeSAMDC*	EF550528	SAMDC	F5’- GACTTGCCAGTTTCTGCCA -3’R5′- CGGACAGCACATAGGAATCAA -3′
*LeSPDS*	NM_001247564	SPDS	F5′- TGGAGGCAGCCAATAACA -3′R5′- CCTTCCCATAAGTTGATGACTG -3′
*LeSPMS*	AY335900	SPMS	F5′- GAGAAGCACATTCCCTGAAAG -3′R5′- AGAACTCCACCATCACCACC -3′
*LeNR*	HQ616893	NR	F5′-ATCACCCAGAGAAGCCAACA-3′R5′-GAGGGTCTCATCGGTAGCTC-3′
*LeNOS1*	XM_004235117	NOS	F5′-GAGCTCCGTTACACACATCG-3′R5′-CGACACCGTCCACAAAGAAT-3′
*LeNCED1*	AJ439079.2	NCED	F5′-GAACTTCGTCGTCATTCCTG-3′R5′- CATCTTTCGCGTACTTATCCA-3′
Actin	Q96483	Reference gene	F5′- GAGAAGCACATTCCCTGAAAG -3′R5′- AGAACTCCACCATCACCACC -3′


### Statistical Analysis

Two independent experiments were performed, with three replicates for each treatment. Data were analyzed using Duncan’s multiple range test at a 0.05 significance level. The charts were compiled using the Origin program (version 8.0).

## Results

### Effects of Exogenous PAs on Endogenous NO Biosynthesis Pathway in Tomato Leaves under Chilling Stress

**FIGURE 1:**
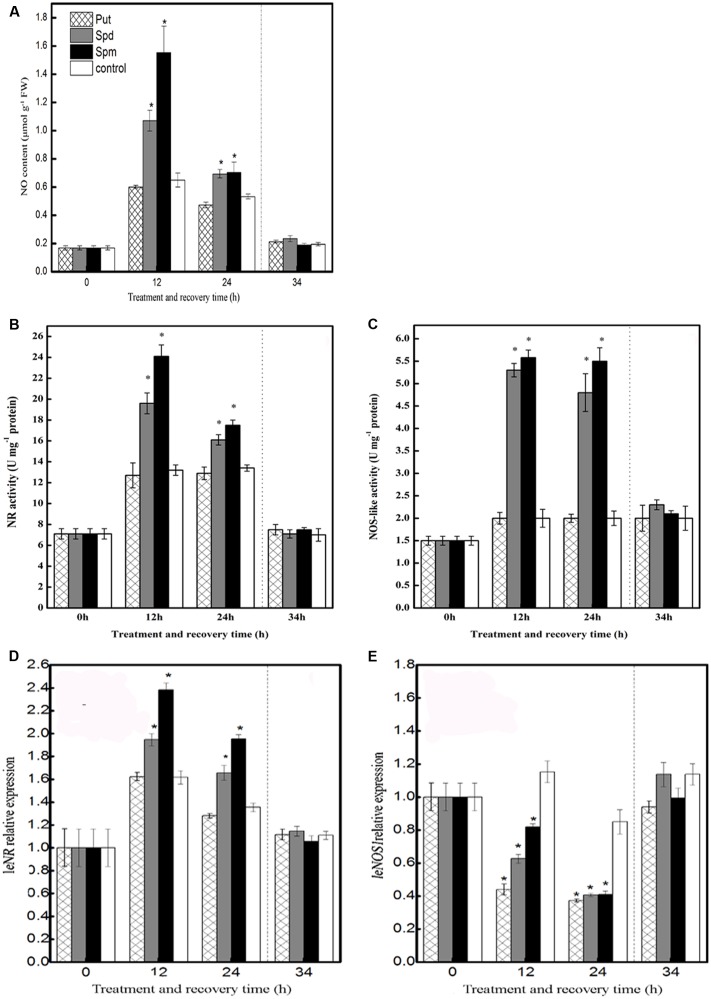
**Effect of exogenous PAs on NO biosynthesis pathway.** **(A)** NO content, **(B)** NR activity, **(C)** NOS-like activity, **(D)** *NR* relative expression, and **(E)** *NOS1* relative expression in the leaves of tomato under chilling stress and recovery period. Seedlings were treated with PAs (1 mM Put, 1 mM Spd, and 1 mM Spm) or distilled water (control). The samples were harvested for analysis of NO content during chilling stress (4°C; 0, 12, and 24 h) and recovery treatment (25°C; 10 h). Data represent the mean ± SE of three independent experiments and asterisks represent significant differences compared to control at *P* ≤ 0.05 according to Duncan’s multiple range test.

As shown in **Figure 1A**, in control seedlings, chilling stress induced a slight release of NO at 12 and 24 h; however, after the recovery treatment, this effect was not observed. Compared to the control, treatment with Put did not induce any obvious change in NO accumulation throughout the treatment period. In contrast, exogenous application of Spd or Spm induced a significantly higher production of NO at 12 h and 24 h of chilling stress than in the control, but this effect was not sustained following the recovery period (**Figure 1A**).

Nitrite reductase activity increased under chilling stress both in control and in Put-treated seedlings, with no significant difference between them. However, chilling-stressed plants that had been treated with Spd showed a dramatic increase in NR activity relative to control seedlings after 12 and 24 h of chilling stress. Similar results were observed for Spm treatment. None of the three PA treatments appreciably altered the NR activity relative to the control during the recovery time, however (**Figure 1B**).

Nitric oxide synthase-like activity was not induced by Put treatment, relative to the control, at any time during the entire treatment and recovery period. In Spd- and Spm-treated plants, on the other hand, NOS-like activity was significantly increased, relative to the control, after 12 and 24 h of stress treatment. None of the PAs had any effect on the levels of NOS-like activity during the recovery time (**Figure 1C**).

In tomato, NR and NOS-like activities are encoded by the genes *LeNR* and *LeNOS1*, respectively. In the present study, compared to the control, exogenous Put did not alter *LeNR* transcript levels throughout the period of treatment. In contrast, Spd treatment increased *LeNR* transcript levels significantly after 12 and 24 h of chilling stress, compared to the levels in the control, and the transcript levels peaked at 12 h of the treatment. Thereafter, however, *LeNR* expression during the recovery period was scarcely affected relative to the control. The effects of Spm were similar to those of Spd; again, no significant change was observed in the level of *LeNR* transcript during the recovery period (**Figure 1D**).

In the case of *LeNOS1*, the application of Put, Spd, or Spm reduced expression, relative to the control. during the chilling-stress period; however, expression during the subsequent recovery period was not appreciably affected (**Figure 1E**).

### Effects of Exogenous PAs on Endogenous H_2_O_2_ Production and PAs Degradative Enzymes Activities in Tomato Leaves under Chilling Stress

**FIGURE 2:**
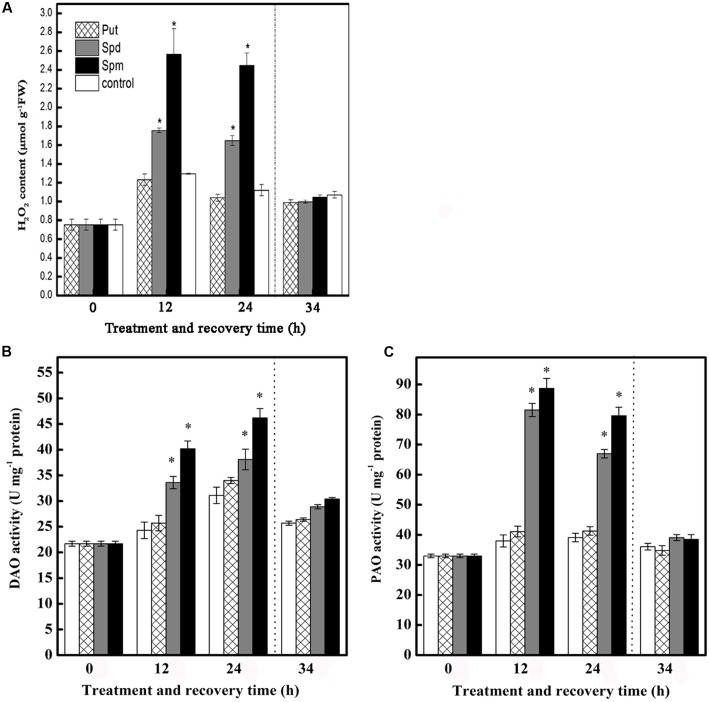
**Effect of exogenous PAs on** **(A)** H_2_O_2_ content, **(B)** DAO activity, and **(C)** PAO activity in the leaves of tomato under chilling stress. The treatment details are as in the **Figure 1**. Data represent the mean ± SE of three independent experiments and asterisks represent significant differences compared to control at *P* ≤ 0.05 according to Duncan’s multiple range test.

Chilling stress induced H_2_O_2_ accumulation at 12 and 24 h in control seedlings, though this effect did not persist during the subsequent recovery period. This accumulation of H_2_O_2_ at 12 and 24 h was greatly increased in seedlings treated with Spd or Spm, but not in seedlings treated with Put. None of the three PAs had any significant effect on H_2_O_2_ levels during the subsequent recovery period (**Figure 2A**).

Seedlings treated with Spd and Spm showed significantly increased activities of DAO and PAO, relative to control seedlings, at 12 and 24 h of stress treatment, but this effect did not persist into the recovery period. Treatment with Put barely had essentially no effect on DAO and PAO activities throughout the treatment period (**Figures 2B,C**).

### Effects of H_2_O_2_ Inhibitor and Scavenger on Spd-induced NO Production in Tomato Leaves under Chilling Stress

**FIGURE 3:**
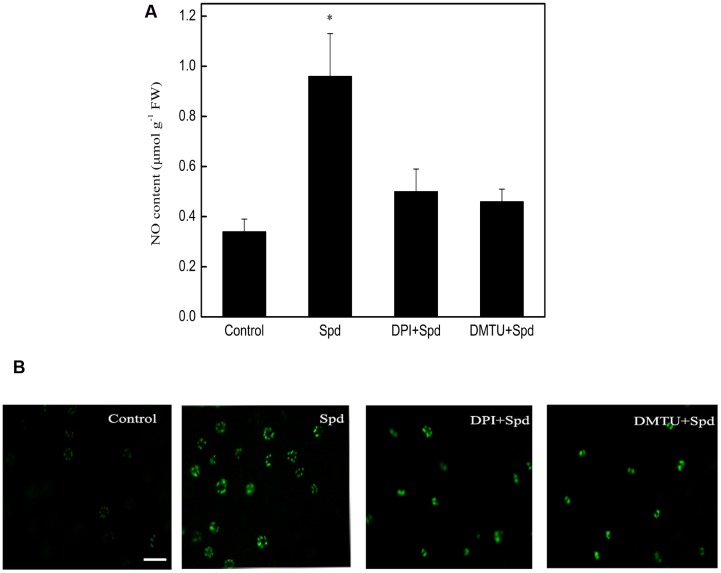
**Effect of DPI and DMTU on Spd-induced NO production in the leaves of tomato under chilling stress (4°C, 24 h).** **(A)** NO content, **(B)** fluorescence imagines of NO. Scale bar for NO accumulation represents 60 μM. Four treatments were used: (1) control; (2) 1 mM Spd; (3) 100 μM DPI+1 mM Spd; (4) 5 mM DMTU+1 mM Spd. Data represent the mean ± SE of three independent experiments and asterisks represent significant differences compared to control at *P* ≤ 0.05 according to Duncan’s multiple range test.

Both the fluorescence detection of NO and its spectrophotometric determination suggested that, relative to control seedlings, treatment with Spd significantly induced NO accumulation during subsequent chilling stress. Compared to treatment with Spd alone, treatment with Spd together with DPI (a NADPH oxidase inhibitor) and DMTU (a H_2_O_2_ and OH^•^ scavenger) greatly reduced the effect of Spd (**Figures 3A,B**), suggesting that H_2_O_2_ can participate in Spd-induced NO production during chilling stress.

### Effect of NO Inhibitor and Scavenger on Spd-induced H_2_O_2_ Generation in Tomato Leaves under Chilling Stress

**FIGURE 4:**
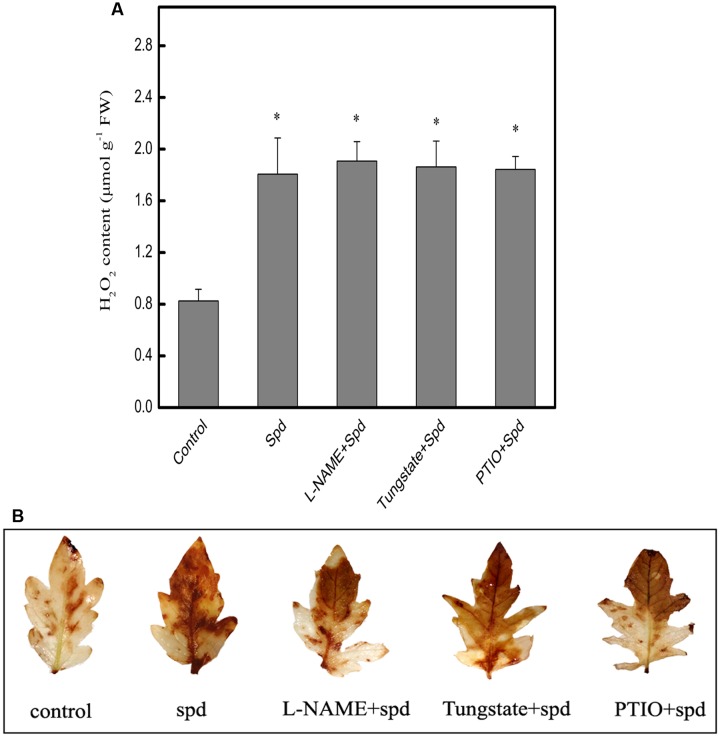
**Effect of different treatments on Spd-induced H_2_O_2_ generation in the leaves of tomato under chilling stress (4°C, 24 h).** **(A)** H_2_O_2_ content, **(B)** detection of H_2_O_2_ by DAB staining. Five treatments were used: (1) control; (2) 1 mM Spd; (3) 200 μM L-NAME+1 mM Spd; (4) 200 μM Tungstate+1 mM Spd; (5) 200 μM PTIO+1 mM Spd. Data represent the mean ± SE of three independent experiments and asterisks represent significant differences compared to control at *P* ≤ 0.05 according to Duncan’s multiple range test.

As shown in **Figure 4A**, treatment with Spd dramatically enhanced the H_2_O_2_ content in chilling-treated seedlings. In contrast, when tungstate (an inhibitor of NR), L-NAME (an inhibitor of NOS), and PTIO (a scavenger of NO) were applied in advance of the Spd treatment, H_2_O_2_ levels were barely increased. This finding was confirmed histochemically using DAB staining for detection of H_2_O_2_ (**Figure 4B**).

### Effects of Exogenous NO on Free Endogenous PAs Contents in Tomato Leaves under Chilling Stress

**FIGURE 5:**
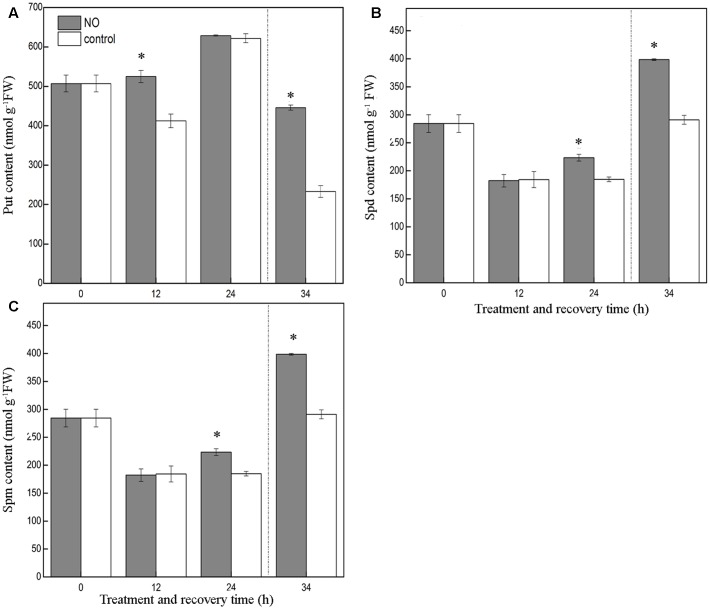
**Effect of exogenous NO on PAs content in the leaves of tomato under chilling stress.** **(A)** Put content, **(B)** Spd content, **(C)** Spm content. Tomato seedlings were treated with 200 μM SNP or distilled water (control), and exposed to chilling stress at 4°C (0, 12, and 24 h), and recovery treatment (25°C; 10 h). Data represent the mean ± SE of three independent experiments and asterisks represent significant differences compared to control at *P* ≤ 0.05 according to Duncan’s multiple range test.

As shown in **Figure 5A**, application of SNP led to a dramatic increase in leaf free Put content at 12 h of chilling stress, relative to control seedlings, but no obvious effect was detected at 24 h. However, during the recovery period, the leaves of seedlings treated with SNP showed a free Put content that was 91.5% higher than that of control leaves.

Compared to the control, SNP had no pronounced effect on free Spd content at 12 h of chilling stress; however, at 24 h and during the recovery period, SNP clearly enhanced free Spd content, relative to the control values, by about 20.9 and 36.9%, respectively (**Figure 5B**).

In contrast to its effects on free Put and free Spd, SNP barely changed the free Spm content throughout the treatment period, indicating that exogenous SNP had little effect on free Spm levels (**Figure 5C**).

### Effects of Exogenous NO on Gene Expressions of PAs Biosynthetic Enzymes in Tomato Leaves under Chilling Stress

**FIGURE 6:**
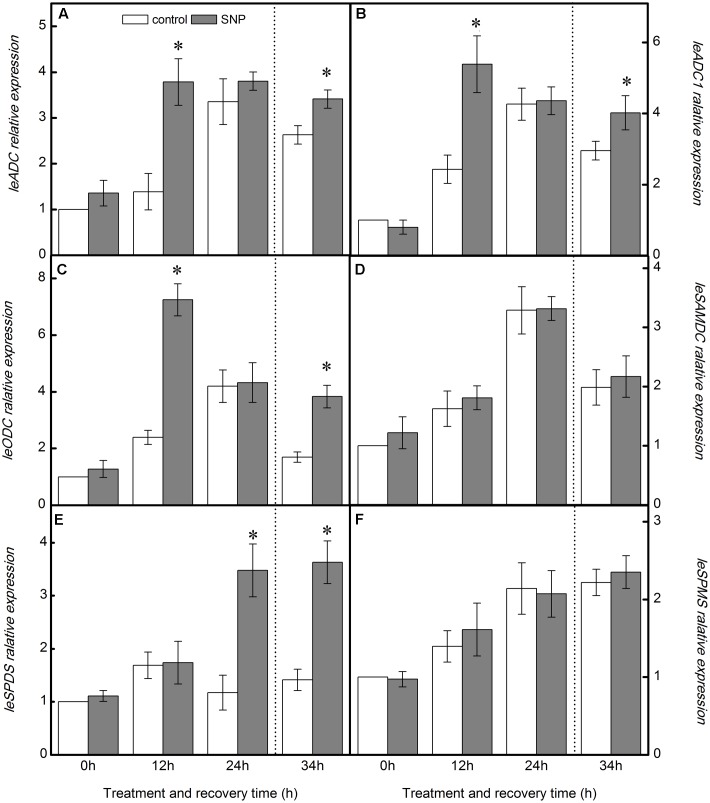
**Effect of exogenous NO on PAs biosynthetic enzyme genes relative expression in the leaves of tomato under chilling stress.** **(A)** *LeADC* relative expression, **(B)** *LeADC1* relative expression, **(C)** *LeODC* relative expression, **(D)** *LeSAMDC* relative expression, **(E)** *LeSPDS* relative expression, and **(F)** *LeSPMS* relative expression. The treatment details are as in the **Figure 5**. Data represent the mean ± SE of three independent experiments and asterisks represent significant differences compared to control at *P* ≤ 0.05 according to Duncan’s multiple range test.

As shown in **Figure 6**, application of SNP significantly increased the expression of *LeODC. LeADC*, and *LeADC1* at 12 h of chilling-stress treatment, relative to control seedlings, but had little effect on *LeSAMDC, LeSPDS*, and *LeSPMS* transcript levels. With the exception of an increase in *LeSPDS* expression, similar results were obtained at 24 h. On the other hand, during the recovery period, exogenous SNP upregulated the expression of *LeODC. LeADC. LeADC1*, and *LeSPDS*, but scarcely affected the expression of *LeSAMDC and LeSPMS* (**Figure 6**).

### Effects of Exogenous Put and D-Arg on ABA Content in Tomato Leaves under Chilling Stress

**FIGURE 7:**
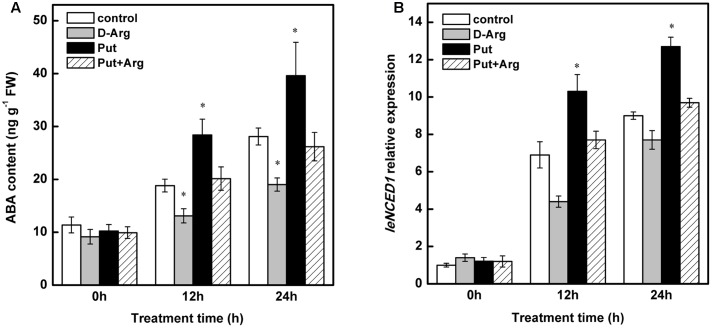
**Effect of exogenous Put and D-Arg on ABA content and *NCED1* relative expression in the leaves of tomato under chilling stress.** **(A)** ABA content, **(B)** *NCED1* relative expression. Four treatments were used: (1) control; (2) 1 mM D-Arg; (3) 1 mM Put; (4) 1 mM D-Arg+ 1 mM Put. Seedlings were exposed to chilling stress at 4°C for 24 h. Data represent the mean ± SE of three independent experiments and asterisks represent significant differences compared to control at *P* ≤ 0.05 according to Duncan’s multiple range test.

As shown in **Figure 7A**, In control seedlings, chilling stress significantly increased the leaf ABA content at both 12 and 24 h. This effect was increased in Put-treated seedlings. This effect of Put was substantially reduced by treatment with D-Arg (Put biosynthesis inhibitor). A significant decrease in ABA levels, relative to control seedlings, was observed during chilling stress in D-Arg-treated seedlings.

As shown in **Figure 7B**, increased *LeNCED1* expression was observed in control seedlings under chilling stress. In Put-treated seedlings, this effect was greatly enhanced. In contrast, treatment with D-Arg reduced the expression of *LeNCED1* relative to that in control seedlings. This effect of D-Arg was offset in Put-treated seedlings.

### Effects of Exogenous ABA and D-Arg on Electrolyte Leakage in Tomato Leaves under Chilling Stress

**FIGURE 8:**
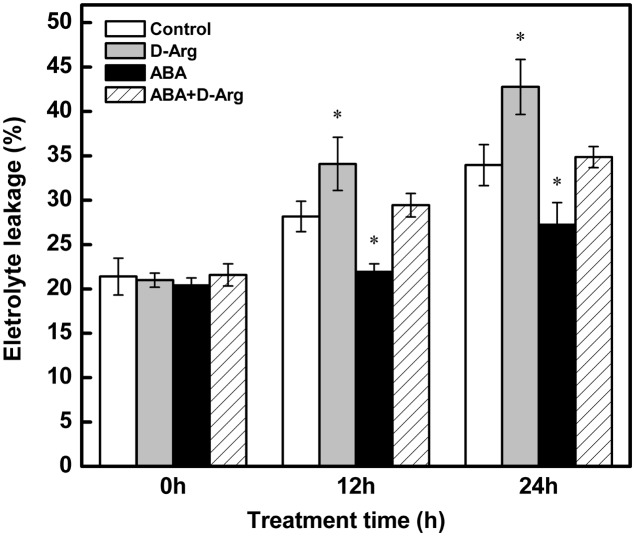
**Electrolyte Leakage of different treatments in tomato leaves during chilling stress.** Four treatments were used: (1) control; (2) 1 mM D-Arg; (3) 1 mM ABA; (4) 1 mM D-Arg+ 1 mM ABA. Seedlings were exposed to chilling stress at 4°C for 24 h. Data represent the mean ± SE of three independent experiments and asterisks represent significant differences compared to control at *P* ≤ 0.05 according to Duncan’s multiple range test.

Compared to the control, chilling stress markedly increased EL. Application of ABA dramatically decreased this effect, whereas in contrast. Treatment with D-Arg enhanced it. The effect of D-Arg could be offset by the application of ABA (**Figure 8**).

## Discussion

It is well established that NO is a bioactive signaling molecule that is widespread in living organisms. Not only is NO involved in the regulation of plant growth and development, but it also plays an important role in the response of plants to abiotic and biotic stresses (García-Mata and Lamattina, 2001; Neill et al., 2003; Zhao et al., 2004). In some cases, it is probably associated with an antioxidative function (Lamattina et al., 2003; Laspina et al., 2005). It is also likely that the protective effects of NO can be achieved through influencing the expression of defense-related genes and the defense signaling cascade (Neill et al., 2003). Besides the interplay of NO with other signals, NO is also linked with phytohormones (Huang et al., 2004; Zhou et al., 2005). For example, Zhang et al. (2007) indicated that NO participates in enhancing the ABA-induced antioxidant capability in maize. Moreover, because of the common precursor L-arginine, NO has a connection with PAs. Results obtained in *Arabidopsis* revealed that the NO induced by PAs had a key role in alleviating stress injury (Tun et al., 2006). To elucidate the impact of exogenous PAs on NO production in tomato seedlings under chilling stress, we monitored the NO content in leaves. Our results showed that, throughout the treatment period, the application of Put had no clear effect on NO accumulation. In contrast, both Spd and Spm treatment significantly enhanced NO production under chilling stress, although no obvious difference relative to the control was observed during the subsequent recovery period (**Figure 1A**). We, therefore, suggest that exogenous Spd and Spm increased NO production under chilling stress. Analogously, Arasimowicz-Jelonek et al. (2009) suggested that Spd and Spm induced NO release in cucumber leaves under drought stress. Similar results were also observed in other studies (Groppa et al., 2008; Gong et al., 2014; Peng et al., 2015). However, Silveira et al. (2006) and Yang et al. (2014) suggested that exogenous Put greatly induced NO production in *Araucaria angustifolia* and in soybean. The contrasting results could be because the effect of PAs on NO production varied with the plant species, the type of PA, and the particular stress. NO production induced by PAs might be correlated with other physiological processes; for example, Santa-Catarina et al. (2007) observed that PA-induced NO plays a pivotal function in embryogenesis and Gong et al. (2014) reported that PA-induced NO production is beneficial in enhancing alkaline-stress tolerance. Similarly, in our previous studies, NO production was observed during Spd-induced chilling tolerance in tomato seedlings (Diao et al., 2016). Therefore, we speculate that in tomato seedlings either Spd or Spm may enhance chilling tolerance by inducing NO production.

Evidence obtained by Tun et al. (2006) showed that PAs induced NO production probably via an enzymatic pathway. Two potential sources of NO production, the NOS-like and NR enzymatic pathways, have been documented in plants (Desikan et al., 2002; Guo et al., 2003). In the present study, Spd and Spm induced NO generation through NR and NOS-like pathway in tomato seedlings under chilling stress (**Figures 1B,C**). Similar results were also obtained by Arasimowicz-Jelonek et al. (2009) in cucumber leaves during drought stress. There are several potential sources of NO generation in plants; however, the existence of any other enzymatic pathway that can directly lead to the PA-induced production of NO is unknown, and further studies are needed in this regard. In the present study, we assessed the transcript levels of *LeNR* and *LeNOS1*, which encode NR and NOS-like activity, respectively. The application of Spd and Spm increased the transcript levels of *LeNR* but reduced those of *LeNOS1* under chilling stress (**Figures 1D,E**). The present results are in agreement with recent studies that indicate a principal role for NR in NO production (Gupta et al., 2011; Ziogas et al., 2015). However, these recent studies are inconsistent with regards to NO generation, most probably due to uncertainty concerning the genes involved. There have been few studies involving the two genes, and in most cases, the involvement of the genes has been predicted rather than demonstrated directly. Further clarification is therefore required.

Polyamines-induced increase in H_2_O_2_ generation is closely linked to Put, Spd, or Spm catabolism (Wimalasekera et al., 2011a). It has been reported that low H_2_O_2_ levels serve as a signal in the signal transduction network of biotic and abiotic stresses (Tanou et al., 2009; Jiang et al., 2012), whereas high H_2_O_2_ levels can cause extensive cell injury or death (Quan et al., 2008). The present study showed that both Spd and Spm treatment, but not Put treatment, caused a large increase in the level of H_2_O_2_ compared to that in the control (**Figure 2A**). Furthermore, a significant increase in PAO activity was induced by the exogenous application of Spd or Spm, potential substrates for H_2_O_2_ generation (**Figure 2C**). Conversely, the application of Put had little effect on H_2_O_2_ content and induced no change in DAO activity. Similar results were obtained by Tanou et al. (2014) in citrus, and by Yoda et al. (2006), Moschou et al. (2008), and Iannone et al. (2013) in tobacco. Therefore, the present study strongly suggested that H_2_O_2_ production induced by Spd or Spm is related to increased PAO activity.

Hydrogen peroxide and NO are signaling molecules; their generation often occurs in short bursts, one after the other or in parallel, and they can exert their influence synergistically or independently (Bright et al., 2006; Pasqualini et al., 2009). In our previous study, Spm showed little correlation with chilling resistance in tomato seedlings (Song et al., 2014a). Therefore, in the present study, we focused on the effect of Spd on the relationship between H_2_O_2_ and NO under chilling stress. We examined H_2_O_2_ and NO contents in plants treated with inhibitors and scavengers of NO and H_2_O_2_, prior to being treated with Spd. As shown in **Figure 3**, application of DPI and DMTU caused a decline in H_2_O_2_ content compared to plants treated with Spd alone (**Supplementary Figure S1**). Similarly, application of DPI and DMTU substantially repressed the Spd-induced increase in NO levels. In contrast, application of L-NAME, tungstate, and PTIO barely affected H_2_O_2_ levels when compared to the levels with Spd treatment alone (**Figure 4**). Thus, these results indicate that in tomato seedlings H_2_O_2_ may act upstream of NO to enhance its production under chilling stress. Similar effects were also found in *Arabidopsis* (Bright et al., 2006) and in maize leaves (Zhang et al., 2007). However, Pasqualini et al. (2009) indicated that NO treatment could increase H_2_O_2_ levels, whereas NO production showed little effect in response to H_2_O_2_ treatment in tobacco. Overall, it may be concluded that the interplay between NO and H_2_O_2_ in plants is complicated issue to elucidate, and that it depends upon the species, the types of stress, and the experimental conditions.

Several reviews have indicated that various abiotic stresses, including chilling stress, could induce different levels of PAs accumulation (Pang et al., 2007; Groppa and Benavides, 2008). We have also previously demonstrated that Put content increased in tomato seedlings under low-temperature conditions (Song et al., 2014a). To determine whether NO induces the generation of PAs, SNP was applied exogenously as a source of NO. In this experiment, pretreatment with NO led to increased Put levels at 12 h of chilling stress, and to increased Spd levels at 24 h. The levels of both Put and Spd were also higher during the recovery period. In contrast, Spm levels were essentially unchanged throughout (**Figure 5**). As observed recently by Filippou et al. (2013), treatment with SNP led to enhancement of Put levels in *Medicago truncatula plants* plants. In addition, Fan et al. (2013) observed that exogenous SNP enhanced the salt tolerance of cucumber by increasing the (Spd+Spm)/Put ratio. Li et al. (2014) suggested that application of SNP could convert Put into Spd or Spm and confer tolerance to chilling stress. The results in the present study might be due to the direct induction of Put and Spd production by NO via an unknown pathway; however, it is possible that NO induced an increase in Put content alone, and that thereafter Put was converted into Spd.

In the work reported here, treatment with SNP greatly increased the expression of *LeODC. LeADC*, and *LeADC1* at 12 h of chilling stress and during the recovery period (**Figure 6**), consistent with the observed Put accumulation (**Figure 5**). The increased *LeSPDS* expression could be responsible for the increase in the level of Spd at 24 h of chilling stress and during the recovery period. The application of SNP barely had an impact on the expression of *LeSAMDC* and *LeSPMS* (**Figure 6**), which appears consistent with the lack of change in Spm levels throughout the treatment period. The results revealed that exogenous NO might modulate the contents of PAs by influencing PA biosynthetic enzymes at the transcriptional level under chilling stress.

Furthermore, results from our previous research indicated that Put, unlike Spd, plays a crucial role in the tolerance of tomato to chilling stress (Song et al., 2014b). In the present study, application of Put had little effect on NO accumulation (**Figure 1A**); however, it could significantly increase endogenous ABA content and upregulate *LeNCED1* under chilling stress (**Figure 7**). In addition, the increase in EL, induced by D-Arg, could be alleviated by ABA (**Figure 8**). These results suggest that ABA is essential for the Put-induced chilling-stress response. As suggested by Cuevas et al. (2008), Put could regulate ABA in response to low temperature.

**FIGURE 9:**
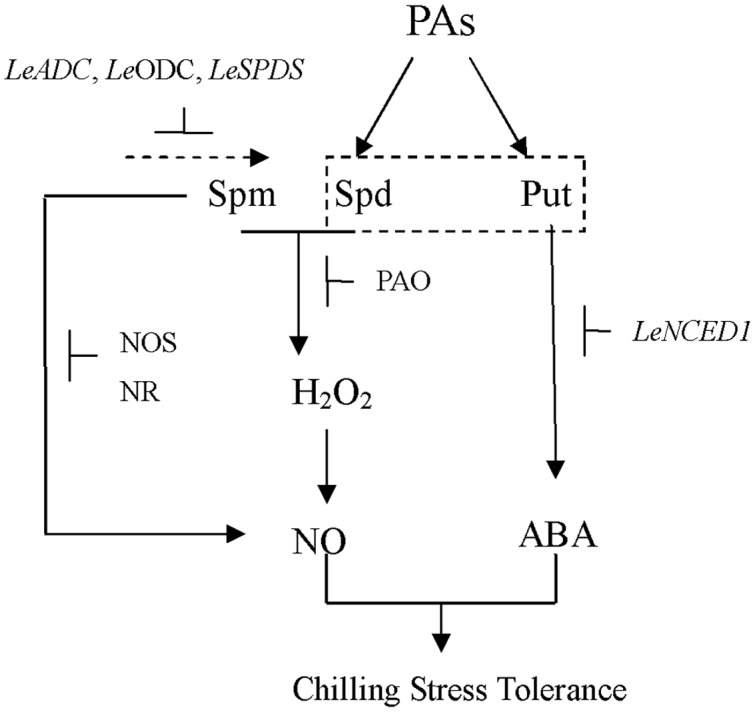
**The model of PAs signaling under chilling stress in tomato seedlings.** Put induces the expression of *LeNCED1*, resulting in the increase of ABA level. Spd and Spm stimulate PAO activity; enhance NR and NOS-like activities, leading to the increase of H_2_O_2_ and NO generation. H_2_O_2_ may act as an upstream signal to stimulate NO production. In turn, SNP (an NO donor) improves Put and Spd contents through up-regulating the expression of *LeADC, LeODC*, and *LeSPDS*. NO and ABA are probably involved in PAs induced chilling stress tolerance.

Data from the present study have revealed the impact of exogenous PAs on NO and H_2_O_2_ (**Figure 9**). In addition, it appears that H_2_O_2_ might act upstream of NO in Spd-treated tomato leaves. These results clearly suggest that signaling involving PAs could correlate with other signaling intermediates. Based on the results of this and previous studies, we suggest that exogenous Spd could increase the chilling tolerance of tomato seedlings by inducing NO biosynthesis under chilling stress. Furthermore, the results of our previous study demonstrated that Put plays important roles in the chilling-stress tolerance of tomato seedlings. Hence, in the present study, the roles of Put and Spd in alleviating chilling stress were of particular interest. The results presented here suggest that Put may increase ABA content by inducing the expression of *LeNCED1*, thereby inducing chilling tolerance. we therefore suspect that cross-talk between PAs and ABA, NO, and H_2_O_2_ is involved in the response of tomato seedlings to chilling stress.

## Conclusion

From the data obtained in this study, it is concluded that a link exists between PAs and NO during chilling stress in tomato seedlings. The exogenous application of Spd and Spm induced the generation of NO in an H_2_O_2_-dependent manner by NOS-like and NR pathways. Put could improve chilling tolerance via activation of ABA synthesis. Furthermore, under conditions of chilling stress, the application of NO enhanced endogenous Put and Spd levels through upregulation of the relevant PA biosynthetic genes.

## Author Contributions

QD and HQ designed research. QD and YS performed research. QD analyzed data. QD and HQ wrote the paper. YS and DS helped to revise the paper.

## Conflict of Interest Statement

The authors declare that the research was conducted in the absence of any commercial or financial relationships that could be construed as a potential conflict of interest.

## Supplementary Material

**FIGURE S1 | Effect of DPI and DMTU on Spd-induced H_2_O_2_ production in the leaves of tomato under chilling stress (4°C, 24 h).** **(A)** H_2_O_2_ content, **(B)** detection of H_2_O_2_ by DAB staining. Four treatments were used: (1) control; (2) 1 mM Spd; (3) 100 μM DPI +1 mM Spd; (4) 5 mM DMTU+1 mM Spd. Data represent the mean ± SE of three independent experiments and asterisks represent significant differences compared to control at *P* ≤ 0.05 according to Duncan’s multiple range test.

## Abbreviations

ABA: abscisic acid
ADC: arginine decarboxylase
BR: brassinosteroid
DAO: diamine oxidase
D-Arg: D-arginine
DMTU: dimethylthiourea
DPI: diphenyleneodonium
EL: electrolyte leakage
GABA: gamma-aminobutyric acid
H_2_O_2_: hydrogen peroxide
L-NAME: N^G^-nitro-L-Arg methyl ester
NCED: 9-cis-Epoxycarotenoid dioxygenase
NO: nitric oxide
NOS: nitric oxide synthase
NR: nitrite reductase
ODC: ornithine decarboxylase
PAs: polyamines
PAO: polyamine oxidase
PMSF: phenylmethylsulfonyl fluoride
PSII: photosystem II
PTIO: 2-phenyl-4,4,5,5-tetramethylimidazoline-1-oxyl-3-oxide
Put: putrescine
PVPP: polyvinyl polypyrrolidone
ROS: reactive oxygen species
SA: salicylic acid
SAM: S-Adenosylmethionine
SAMDC: S-Adenosylmethionine decarboxylase
SNP: sodium nitroprusside
Spd: spermidine
SPDS: spd synthase
Spm: spermine
SPMS: spm synthase
