# Supplementary figures and images for: Interaction of Polyamines, Abscisic Acid, Nitric Oxide, and Hydrogen Peroxide under Chilling Stress in Tomato (Lycopersicon esculentum Mill.) Seedlings
Source: Front Plant Sci. 2017 Feb 14;8:203. doi: 10.3389/fpls.2017.00203 (PMC5306283; doi:10.3389/fpls.2017.00203)

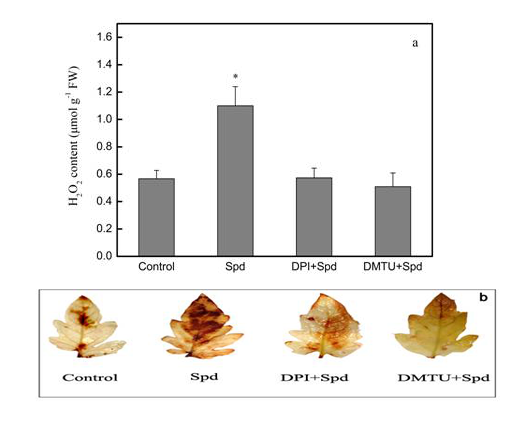

Supplement: Supplementary file 1 [file Image_1.TIF]
